# Evaluation of Disease Severity and Global Transcriptome Response Induced by *Citrus bark cracking viroid*, *Hop latent viroid*, and Their Co-Infection in Hop (*Humulus lupulus* L.)

**DOI:** 10.3390/ijms20133154

**Published:** 2019-06-28

**Authors:** Nataša Štajner, Sebastjan Radišek, Ajay Kumar Mishra, Vishnu Sukumari Nath, Jaroslav Matoušek, Jernej Jakše

**Affiliations:** 1 University of Ljubljana, Biotechnical Faculty, Department of Agronomy, Jamnikarjeva 101, SI-1000 Ljubljana, Slovenia; 2 Slovenian Institute of Hop Research and Brewing, Plant Protection Department, Cesta Žalskega tabora 2, SI-3310 Žalec, Slovenia; 3 Biology Centre of the Czech Academy of Sciences, Institute of Plant Molecular Biology, Department of Molecular Genetics, Branišovská 31, 37005 České Budějovice, Czech Republic

**Keywords:** *Citrus bark cracking viroid*, co-infection, differentially expressed genes, *Humulus lupulus*, *Hop latent viroid*, transcriptome profiling

## Abstract

Viroids are small non-capsidated, single-stranded, covalently-closed circular noncoding RNA replicons of 239–401 nucleotides that exploit host factors for their replication, and some cause disease in several economically important crop plants, while others appear to be benign. The proposed mechanisms of viroid pathogenesis include direct interaction of the genomic viroid RNA with host factors and post-transcriptional or transcriptional gene silencing via viroid-derived small RNAs (vd-sRNAs) generated by the host defensive machinery. *Humulus lupulus* (hop) plants are hosts to several viroids among which *Hop latent viroid* (HLVd) and *Citrus bark cracking viroid* (CBCVd) are attractive model systems for the study of viroid-host interactions due to the symptomless infection of the former and severe symptoms induced by the latter in this indicator host. To better understand their interactions with hop plant, a comparative transcriptomic analysis based on RNA sequencing (RNA-seq) was performed to reveal the transcriptional alterations induced as a result of single HLVd and CBCVd infection in hop. Additionally, the effect of HLVd on the aggressiveness of CBCVd that underlies severe stunting in hop in a mixed infection was studied by transcriptomic analysis. Our analysis revealed that CBCVd infection resulted in dynamic changes in the activity of genes as compared to single HLVd infection and their mixed infection. The differentially expressed genes that are involved in defense, phytohormone signaling, photosynthesis and chloroplasts, RNA regulation, processing and binding; protein metabolism and modification; and other mechanisms were more modulated in the CBCVd infection of hop. Nevertheless, Gene Ontology (GO) classification and pathway enrichment analysis showed that the expression of genes involved in the proteolysis mechanism is more active in a mixed infection as compared to a single one, suggesting co-infecting viroids may result in interference with host factors more prominently. Collectively, our results provide a deep transcriptome of hop and insight into complex single HLVd, CBCVd, and their coinfection in hop-plant interactions

## 1. Introduction

Viroids are the smallest known non-encapsidated infectious pathogens, consisting of covalently closed single-stranded RNA molecules ranging in size from 239 to 401 nucleotides (nt), depending on the particular viroid species [1]. Intriguingly, viroids lack a protein-encoding capacity but possess the essential biological information for host specificity, autonomous replication by rolling-circle mechanism, systematic spreading, subcellular localization, and interaction with host factors [2]. Nevertheless, viroids (chloroplastic) possess high rates of in vivo mutation that are orders of magnitude higher than any other nucleic acid-based replication entity, and even single nucleotide substitution can dramatically alter its virulence and host range [3]. These attributes make them enticing models for the study of RNA structure-function relationships. Viroids are cosmopolitan in distribution, and their hosts include monocots and dicots, herbaceous and woody plants, crops, and ornamental plants, which reflects their high potentiality of transmissibility [4]. Viroid-induced symptoms range from necrosis to less severe developmental disorders, including chlorosis, epinasty, leaf deformation and necrosis, stunting, fruit distortion, and plant death [5]. Currently known viroid species are classified into two families depending on their molecular and biological properties: *Pospiviroidae* and *Avsunviroidae*. Members of *Pospiviroidae* are replicated through an asymmetric rolling-circle mechanism in the nucleus, using host DNA-dependent RNA polymerase II [6], resulting in the synthesis of oligomeric, greater-than-unit length RNA replicative intermediates (plus and minus single strand and double-stranded RNA) that are processed by host enzymes into mature viroid circles [7]. In contrast, members of the *Avsunviroidae* family are replicated and accumulated through symmetric rolling circles in chloroplasts by using the nuclear-encoded polymerase (NEP), and further, the oligomeric intermediates undergo via an internal hammerhead encoded self-cleavage ability to unit length and are ligated by host enzyme into mature circles [8,9]. However, in contrast to the explosive discovery of the viroid replication mechanism, the molecular mechanism of viroid-induced pathogenesis and host responses are still enigmatic. Emerging evidence shows that viroid-specific small RNAs (vsRNA) accumulate during viroid infection and are involved in transcriptional gene silencing via gene methylation [10], direct interaction with plant proteins or/and by viroid-induced RNA interference (RNAi) based post-transcriptional gene silencing (PTGS) [11].

Studies of plant–viroid interactions have led to new insight into viroid mutual complex interactions in the host plants. In viroid/viroid interactions, multiple viroid species can exhibit an antagonistic (decrease of individual viroid species titers) or synergistic (increase of individual viroid species titers) relationship, and the outcome of co-infection interactions and corresponding host responses determine the health status or magnitude of disease [12]. In the *Pospiviroidae* family, coinfection and several types of interactions were observed for *Potato spindle tuber viroid* (PSTVd), *Citrus exocortis viroid* (CEVd), *Chrysanthemum chlorotic mottle viroid* (CChMVd), *Chrysanthemum stunt viroid* (CSVd), and *Hop stunt viroid* (HSVd), etc. species based on the severity of disease symptoms in host plants [12]. For instance, *Tomato apical stunt viroid* (TASVd) showed an antagonistic interference in CEVd/ PSTVd infected *Solanum jasminoide* plants [13], whereas the titer of *Citrus dwarfing viroid* (CDVd) was enhanced by *Citrus tristeza virus* (CTV) showing a synergistic relationship in Mexican lime [14].

Hop (*Humulus lupulus* L., *Cannabaceae*) is an economically important crop, mainly cultivated in Europe, western Asia, and North America for specific secondary metabolites, which serve as an essential component in the brewing and pharmaceutical industries [15]. Among other diseases, viroid diseases pose a severe threat to hop cone production. Currently, hop plants are known to be the host for four viroid species, namely *Hop stunt viroid* (HSVd) [16], *Apple fruit crinkle viroid* (AFCVd) [17], *Hop latent viroid* (HLVd) [18], and *Citrus bark cracking viroid* (CBCVd) [19]. The infection caused by HLVd has been reported worldwide in hop growing regions [18]. Although HLVd-infected hop plants are symptomless, infection leads to a significant reduction in bitter acids content [20]. HSVd was first discovered in Japanese hop fields with typical symptoms being reported after 3–7 years of infection, which include stunting, leaf curling, small cone formation, and a substantial reduction of alpha-acid content [21,22]. The disease caused by AFCVd is currently restricted to Japanese hop fields, and symptoms caused by this viroid resemble those of HSVd [21]. Among them, the disease caused by CBCVd is the most aggressive, and symptoms appear after one year of infection. Symptoms include severe bine stunting, leaf down curling, a reduction in cone size, dry root rotting in hops after the first dormancy, and complete plant dieback in 3-5 years [19,23]. Viroids represent the rapidly evolving system, and in this context, comprehensive analysis of gene expression in the viroid-infected host is crucial to dissecting the molecular mechanism responsible for viroid pathogenesis and further developing novel and effective strategies to control the disease.

As the research area of the impact of viroids on host transcriptome has rapidly expanded, and previously published studies based on microarray analysis have revealed the altered gene expression patterns in viroid-infected plants, including the induction or suppression of genes involved in defense and stress responses, cell wall structure, chloroplast biogenesis, signaling pathway, symporter activities, and hormone and protein metabolism, among others [24,25,26,27]. High-throughput sequencing (HTS) technologies such as the Illumina or Ion Torrent RNA-Seq approach have provided a powerful platform for the high-resolution characterization of transcriptome and eradicated several problems associated with microarray technologies in terms of the wider dynamic range of detection, precision, reproducibility, cost efficiency, higher specificity, and sensitivity [28]. In the last few decades, transcriptome profiling has been extensively applied to investigate the global gene expression profile in plant-pathogen compatible interactions. Nevertheless, limited transcriptome-based analyses have been conducted in plant-viroid interactions for the system level understanding of host response to viroid infection, which include the transcriptome profiling of PSTVd-infected potato [29] and tomato [30], PLMVd infected peach [31], CBCVd-infected hop [32] and HSVd-infected hop [33]. These studies have illustrated that viroid infection appears to cause an amendment of gene expression mainly involved in defense response, cell wall structure, protein metabolism, phytohormone homeostasis, hormone signaling, photosynthesis, and primary and secondary metabolism.

Incredibly, the presence of HLVd in new hop planting materials has been claimed in major hop growing fields, suggesting that HLVd is widely distributed in virtually all other hop-growing regions worldwide [34], but still, transcriptome changes in response to HLVd in hop have not been reported. HLVd is considered a common denominator in CBCVd-infected hop plants [19], which raises important questions about the interactions between these two viroids and the etiology of the disease, especially because single CBCVd infections of hop are not present in nature or hop fields. In this context, knowledge of the impact of potential interaction of HLVd and CBCVd with hop immune responses is essential for the development of disease management strategies and the mechanisms that underlie severe stunting in hop in the mixed infection of HLVd and CBCVd.

In this study, we employed comparative transcriptome profiling to investigate genome-wide changes in gene expression associated with individual HLVd and CBCVd hop infections. Furthermore, we have extended our study to investigate global changes in gene expression patterns in the association of HLVd-CBCVd coinfection, which could help in understanding the mechanisms that underlie the induction of more severe disease symptoms in mixed HLVd-CBCVd infection as compared to CBCVd single infection in hop.

## 2. Results

### 2.1. Artificial Viroid Inoculations, Infectivity and Phenotypic Evaluation of Plants

In order to gain preliminary information about differences in the incidence and severity of infections, virus- and viroid-free hop plants were biolistically inoculated with infectious cDNA constructs of single viroid (HLVd, CBCVd) and HLVd-CBCVd combination. The RT-PCR testing in a pre-dormancy period of 4 months post inoculation (mpi) showed viroid infections in 40% of plants inoculated with HLVd, 60% in plants inoculated with CBCVd, whereas in HLVd-CBCVd treatment 30% of plants were infected with both viroids, and the rest of infected coinoculated plants have single HLVd or CBCVd infection (Table S1). Further testing in first (14 mpi) and second (28 mpi) postdormancy periods, revealed increased infection up to 60% for HLVd, up to 80% for CBCVd and 40% for HLVd-CBCVd plants (Table S1), probably as a consequence of mechanical spreading during plant manipulations.

**Figure 1:**
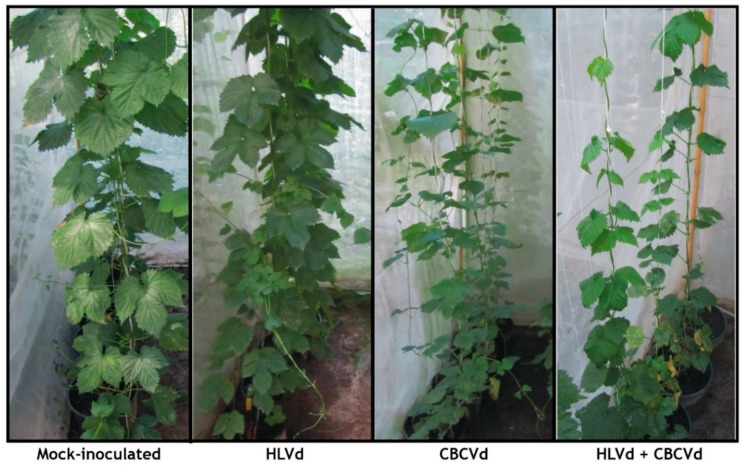
Symptoms induced in hop plants after 28 months of biolistic inoculation of HLVd, CBCVd, and HLVd-CBCVd inoculum. The CBCVd infected and HLVd-CBCVd coinfected plant showing stunted growth, smaller leaves with chlorosis compared to control.

In the predormancy period, hop plants coinfected with HLVd and CBCVd developed some typical symptoms such as leaf down curling and mild yellowing, while plants infected with CBCVd or HLVd were symptomless. After the first dormancy phase, plants infected with CBCVd developed typical leaf deformations, including yellowing of the margins, which were more severe in the double viroid infection. After second dormancy the plant coinfected with HLVd and CBCVd showed a similar trend of disease development with bine cracking and stunted growth but more pronounced as compared to CBCVd infected plants (Table 1; Figure 1). The strand-specific RT-qPCR (ssRT-qPCR) suggested the higher accumulation of minus multimeric strand compared with plus polarity of CBCVd, whereas HLVd exhibited an excess of plus over minus strands in individual infection in the postdormancy period (28 mpi) (Figure S1). Compared to the mock-inoculated control, plants coinfected with HLVd and CBCVd showed a significant biomass reduction (81%) as compared to plants harboring CBCVd (53.7%). These observations confirmed the aggressiveness of CBCVd in causing disease with significant synergistic interaction with HLVd, which in combination caused a more deleterious disease in hop.

### 2.2. NGS Sequencing, De novo Assembly, and Annotation of Transcriptome

To determine the consequences of individual HLVd or CBCVd infection and their interactions with hop at the transcriptional level, total RNA was isolated from mock-inoculated, the biolistically inoculated CBCVd-, HLVd- and CBCVd + HLVd-positive young leaves of hop plants at 28 mpi and samples with RIN > 8 were enriched for mRNA fraction. High throughput sequencing of three biological replicates was performed on Ion Proton NGS platform, which utilizes the detection of hydrogen ions during complementary nucleotide incorporation and offers strand-specific sequencing. Sequencing yielded on average 25.1 M of raw reads, yielding on average 2.69 Gb of raw nucleotide data. Adapter, quality, and length trimming showed that outputs of Ion Torrent contained the high-quality clean reads and numbers were not substantially changed (24.0 M reads and 2.4 Gb). The infectivity of viroids in samples was confirmed by the presence of viroid reads and completely correlated with expected treatments. Quality control parameters indicated that the resulting gene transcript data were reliable. Sequencing results for viroid treatments, controls, and biological replicates are summarized in Table S2. De novo assembly of quality reads, removal of redundancy, and mapping reads back to contigs generated 94,604 unigenes with lengths ranging from 105 to 11,649 bp, with a high percentage of unigenes size of more than 1000 bp (Figure S2A). The cumulative length of the assembly was 53,959,732 bp with N50 and average length values of 681 bp and 570 bp, respectively. The average unigene size was longer than those reported in previous studies, namely, *Spartina alterniflora* (386 bp) [35], chickpea (428 bp) [36], sweet potato (321 bp) [37], tomato (380 bp) [38], etc. The average GC content of hop unigenes was 40.68%, which was in the range of GC contents of coding sequences in dicots. The completeness of the obtained transcriptome was assessed with a BUSCO tool [39] against 1375 single-copy genes from the embryophyta odb9 collection. The comparison showed that transcriptome contains 738 (53.7%) complete single-copy BUSCOs, 411 fragmented BUSCOs (29.9%), while 226 BUSCOs (16.4%) were missing in the assembly.

Functional annotation of unigenes against the NCBI nr protein databases with a significance cut-off E-value of 1.0 E^−3^ revealed that 57,874 (61.17%) assembled unigenes aligned to the nr protein database, while the remaining 36,730 of unigenes (38.83%) did not show significant homology to any available sequence in the database. These sequences might represent the fragmented transcripts, misassemblies, or untranslated parts of the genes. The E-value distribution of the predicted unigenes illustrated that 58.78% of aligned unigenes had an E-value of less than 1.0 E^−50^ and suggested their significant homology with the available gene sequence in the database (Figure S2B). Analysis of the similarity distribution revealed that 54.53% unigenes shared more than 80% sequence length with genes of the nr database (Figure S2C). Species distribution of homology search of unigenes against the nr protein database exhibited that approximately 68.61% of total unigenes were matched to sequences of six top-hit species, namely, *Trema orientalis* (34.04%), *Parasponia andersonii* (18.78%), *Morus notabilis* (8.65%), *Quercus suber* (2.65%), *Vitis vinifera* (2.54%), and *Ziziphus jujube* (1.95%), suggesting significant sequence conservation of hop unigenes with other plant species (Figure S2D).

**Figure 2:**
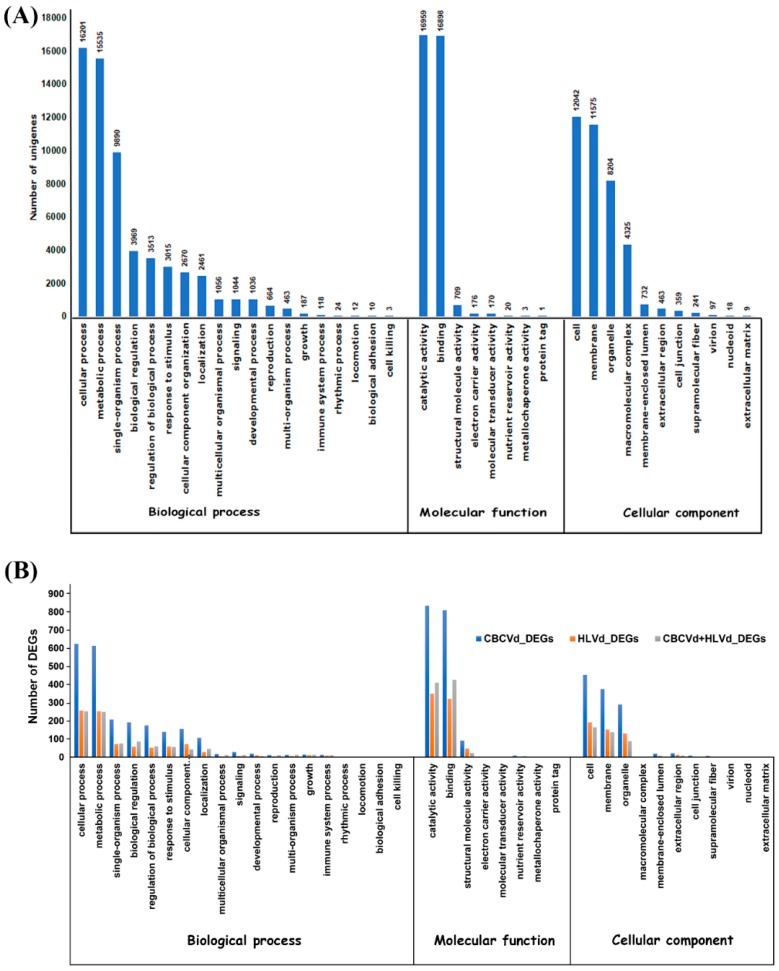
Histogram representation of Gene Ontology (GO) classification annotated transcripts of hop (**A**) and the differentially expressed genes in single CBCVd, HLVd infected, and HLVd-CBCVd coinfected hop (**B**) in three categories: Cellular components, molecular function, and biological processes.

Sequence homology based on GO classification mapped and clustered a total of 40,339 unigenes into three main GO categories, including 38 functional groups (Figure 2A). A total of 1,34,872 GO functional assignments were obtained, among which, biological processes comprised the largest category (61,871, 45.87%), followed by cellular component (38,065, 28.22%) and molecular functions (34,936, 25.90%) (Figure 2A). The alignment of unigenes with the COGs database for orthologous genes clustered 30,405 unigenes into 25 categories based on sequence homology with 31,553 functional annotation due to the multiple COG function of some unigenes (Figure S3). Among the 25 functional categories, the majority of unigenes were associated with “function unknown” (25.97%) followed by “signal transduction mechanism” (12.37%), “post-translational modification, protein turnover, chaperones” (7.72%) and “transcription” (6.21%) (Figure S3).

In order to understand the gene functions with an emphasis on the biochemical pathway, 16,341 KEGG annotated unigenes were categorized into five different functional groups (Table S3). The largest number of unigenes were classified into the “metabolism”, with most of them involved in “carbohydrate metabolism” (13.73%), “amino acid metabolism” (8.45%), “lipid metabolism” (7.70%), “biosynthesis of other secondary metabolites” (5.89%) “energy metabolism” (5.34%), and other sub-categories. Intriguingly, unigenes annotated in the secondary metabolism categories were associated predominantly with terpenoid backbone biosynthesis, prenylflavonoids biosynthesis, flavonoid biosynthesis, sesquiterpenoid and triterpenoid biosynthesis, which was in agreement with our previous report that secondary metabolites are biosynthesized in hop leaves at the detectable limit [40]. In addition to metabolism, unigenes were also classified into “genetic information processing”, which accounted for 4043 KEGG annotated unigenes, most of them involved in “translation” (9.16%), followed by “folding, sorting and degradation” (7.45%), whereas “cellular processes” were represented by 289 KEGG annotated unigenes involved in “transport and catabolism”, “cell growth and death”, “cell motility” and “cell communication”. Additionally, 4,720 unigenes were classified into “environmental information processing (EIP)” and are only involved in “signal transduction” and “membrane transport”.

### 2.3. Validation of High-Throughput Sequencing Data Using RT-qPCR

**Figure 3:**
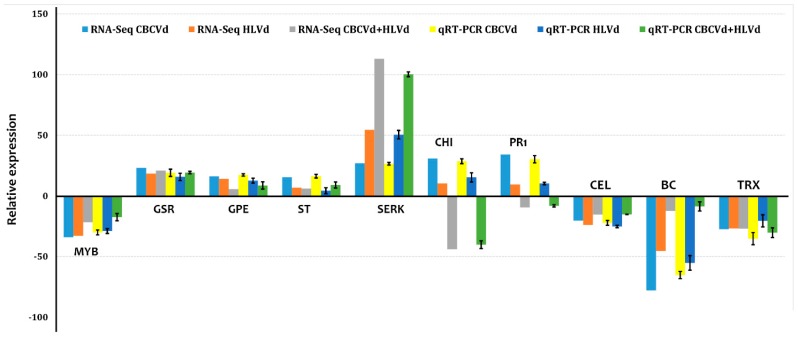
Validation of differentially expressed genes from RNA sequencing by RT-qPCR. Graph showing fold change of genes in a leaf of CBCVd infected HLVd infected, and HLVd-CBCVd coinfected hop. MYB: MYB transcription factor, GSR: Golgi snap receptor complex, GPE: Glucose-6-phosphate 1-epimerase, ST: Bidirectional sugar transport, SERK: Somatic embryogenesis receptor kinase 1, CHI4: Chitinase 4, PR1: Pathogenesis-related protein 1, CEL: Cellulase, BC: Blue copper protein, TRX: Thioredoxin h-type-like. qRT-PCR analyses were normalized using DRH1 (DEAD-box ATPase-RNA-helicase) as an internal control gene. The fold change of each gene was calculated by the 2^−ΔΔCT^ method. The vertical bars indicate standard deviation.

Total RNA samples isolated for RNA sequencing was used to perform RT-qPCR analysis (Figure 3). PCR primers for the amplification of eight randomly chosen highly differentially expressed genes and two selected (PR1 and CH4) were developed from annotated transcripts. DGE analyses showed that genes encoding PR1 and CH4 were significantly downregulated in HLVd-CBCVd coinfection, while upregulated in single CBCVd and HLVd infection in RNA seq profiling. The recent knowledge gleaned from biochemical and molecular studies suggested that PR1 protein plays a major role in plant defense and in compatible host-pathogen interactions over expressions of PR1 protein suppress programmed cell death, thus suggesting their involvement in pathogenesis and symptom expression and the reduction of growth [41]. Similarly, accumulated evidence suggested that chitinase regulate the cell expansion of root and are crucial for normal plant growth and development [42,43]. We, therefore, also employed two of these genes for RT-qPCR analysis, which also showed a similar trend of the significant reduction in mixed HLVd-CBCVd infection compared to a single infection. RT-qPCR analyses with a randomly selected set of eight primer pairs showed a consistent expression profile with six primer pairs as obtained in RNA seq data in all samples. Inconsistencies of expression among the remaining two genes between RT-qPCR and RNA-Seq could be attributed to a lack of specific primers targeting regions with the high discriminatory or plausible difference in sensitivity and normalization factors of the two methods [44]. Nevertheless, the RT-qPCR expression data was in agreement with those obtained from the DGE data, indicating reliable results.

### 2.4. Functional Classification and Comparison of Differentially Expressed Genes

**Figure 4:**
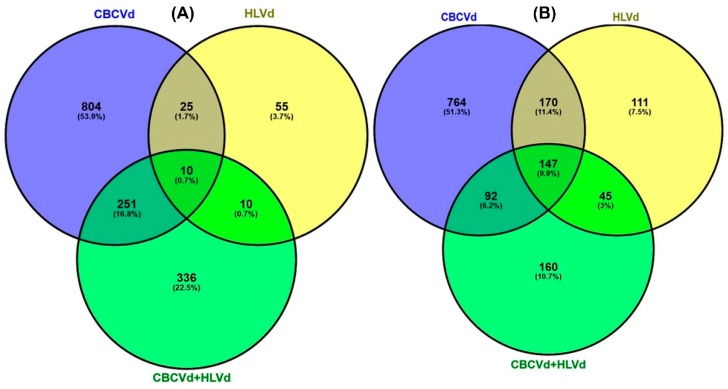
Venn diagrams of upregulated (**A**) and downregulated (**B**) genes identified in single CBCVd, HLVd infected, and HLVd-CBCVd coinfected hop plants.

To obtain comprehensive differential expression patterns among CBCVd, HLVd, and CBCVd- HLVd infected libraries, transcript abundances were calculated and normalized to reads per kb per million reads, which delivers an empirical approach and eliminates bias based on RNA composition [45]. Mapping of all the clean reads onto the non-redundant set of hop transcripts illustrated their range of distribution patterns 0.0 to 2316.18, 0.0 to 7091.90, and 0.00 to 3996.73 (RPKM) in CBCVd (Table S4), HLVd (Table S5), and CBCVd-HLVd (Table S6) libraries, respectively, suggesting the diverse range of change of expression level in single and mixed infection. The comparative transcriptome abundance analysis was carried out between mock-inoculated (viroid free) and viroid infected libraries, which revealed significant differential expression level (*P*-value < 0.05 and logFC ≥ 2 or ≤ –2) of 2,263 [up-regulated (UR): 1090; down-regulated (DR): 1173) and 1,041 (UR: 578; DR: 473) unigenes in individual CBCVd and HLVd-infected hop plants, whereas in CBCVd-HLVd coinfected hop plants 1,051(UR: 607; DR: 444) unigenes were found to be differentially regulated. Strikingly, a smaller number of upregulated (25) and downregulated genes were similar in single CBCVd and HLVd infected hop plants, suggesting the differential alteration pattern of transcriptome (Figure 4).

**Figure 5:**
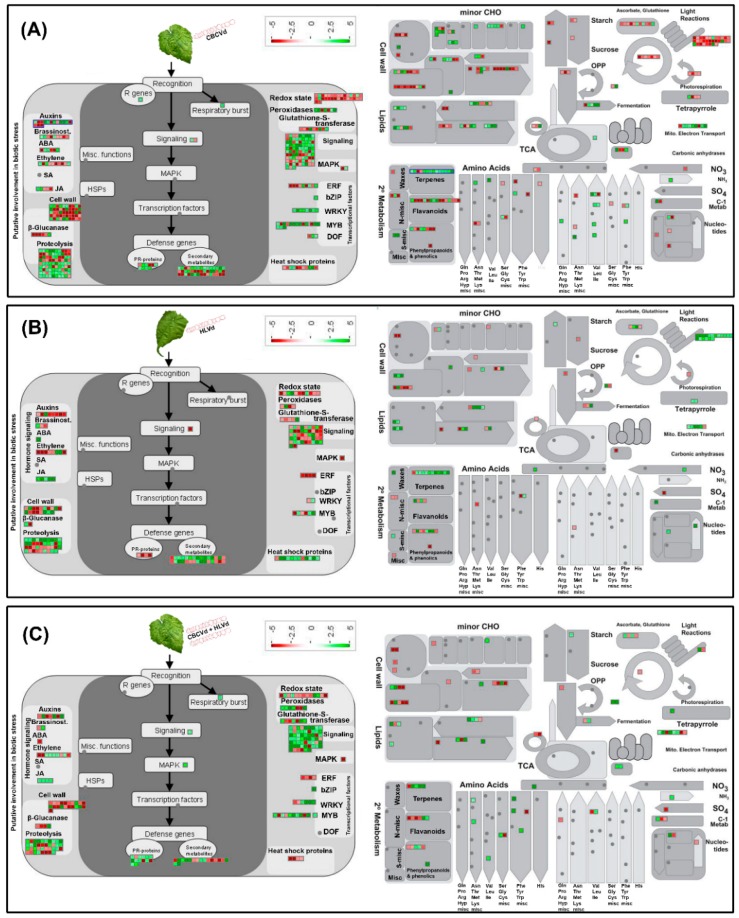
Overview of the MapMan visualization of differences in transcript levels in CBCVd infected (**A**), HLVd infected (**B**), and HLVd-CBCVd coinfected (**C**) hop. The log_2_ fold changes of significantly differentially expressed genes were imported and visualized in MapMan. Red and green displayed signals represent a decrease and an increase in transcript abundance, respectively in single CBCVd, HLVd infected and single CBCVd, HLVd infected, and HLVd-CBCVd coinfected relative to the mock-inoculated samples of hop. The scale used for coloration of the signals (log_2_ ratios) is presented.

**Figure 6:**
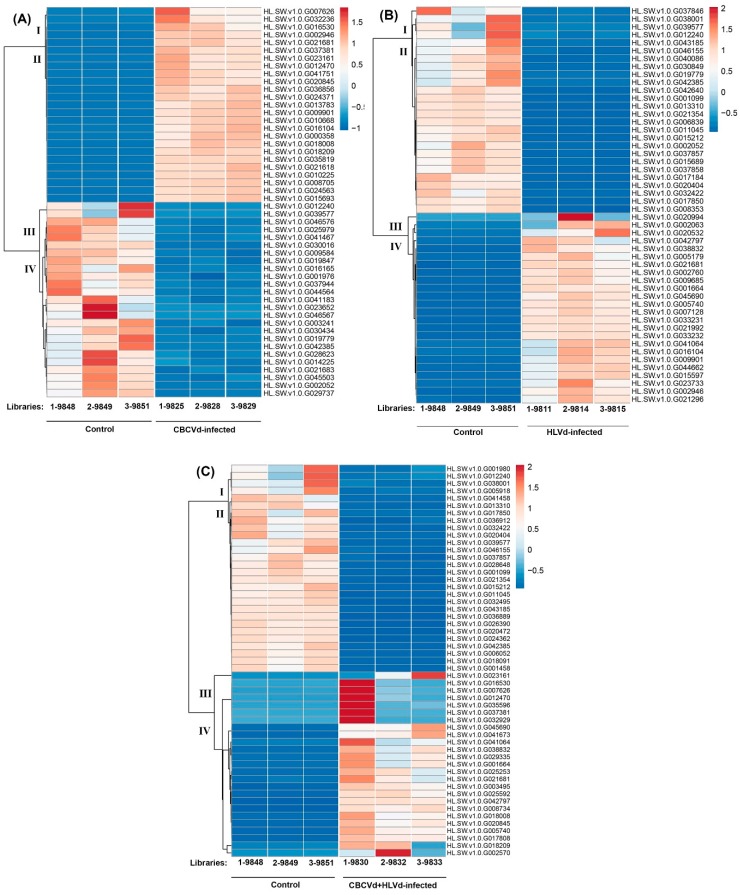
Heat map and complete linkage hierarchical clustering of differentially expressed genes in CBCVd infected (**A**), HLVd infected (**B**) and HLVd-CBCVd coinfected (**C**) hop. Colors on vertical represent the clustered genes based on gene expression, the horizontal line represents the single gene, and color of the line indicates the average gene expression in single and mixed CBCVd, HLVd infection. The signal ratios were shown in a blue-orange-red color scale, where red indicated high expression level and blue indicated low expression level.

Approximately, 98.01% of CBCVd responsive DEGs (UR: 1,063; DR: 1,155), 92.41% of HLVd responsive DEGs (UR: 498; DR: 464), and 98.09% HLVd-CBCVd responsive DEGs (UR: 593; DR: 438) were annotated using the BLASTx procedure against the nr-protein database of NCBI. Furthermore, CBCVd, HLVd, and HLVd-CBCVd responsive DEGs were classified into GO functional categories and further subjected to GO enrichment analysis to find out the correlation between phenotypic differences and gene expression. The GO annotation categorized 1,837 CBCVd responsive DEGs into three classes (16 terms, 41.56%), cellular function (7 terms, 21.96%), and molecular function (5 terms, 31.07%) (Figure 2B). Similarly, the GO annotation categorized 783 HLVd responsive DEGs into three classes (15 terms, 42.07%), cellular function (6 terms, 23.39%), and molecular function (4 terms, 33.89%), whereas GO annotation of HLVd-CBCVd responsive DEGS classified 844 unigenes into 24 subcategorized of three classes (Figure 2B). Moreover, GO functional enrichment analysis provided the overview of statistically significant and relevant GO terms of DEGs, which were modulated in single CBCVd, HLVd, and mixed CBCVd-HLVd infection in hop. To build a biological interpretation, a total of 32 GO terms of CBCVd, HLVd, and CBCVD-HLVd responsive DEGs were screened on account of adjusted *P*-value ≤ 0.05 (Table S7) and their statistically significant enriched GO terms were plotted in two-dimensional scatterplot (Figures S4, S5, S6, S7, S8, and S9). Among the various biological process categories, the GO terms linked to primary metabolic process lipid biosynthesis and cellular process were significantly enriched, whereas in molecular function category GO terms related to catalytic and binding activity, which plays a crucial role in signal recognition and transduction during host-pathogen interaction, were significantly enriched in single CBCVd, HLVd, and mixed HLVd-CBCVd infections. To extend our understanding of specific biological pathways and functions of DEGs, they were subjected to mapping against KEGG database categories according to sequence homology, and the results were compared with the unigenes background. The KEGG pathway analysis revealed that amino acid and lipid metabolism pathways were enhanced in single CBCVd, HLVd, and mixed HLVd-CBCVd infections, whereas equitably large number of unigenes assigned to oxidative phosphorylation and photosynthesis were diminished in CBCVd infection (Table S3), which showed completely opposite expression patterns in HLVd infected plants correlating our phenotypic observation of severe chlorosis in CBCVd infected plants. Intriguingly, we found that the KEGG terms associated to upstream translation pathways such as the mRNA surveillance and ribosome biogenesis pathways were relatively more enriched in HLVd-CBCVd infection compared to their single infection, suggesting that proteolysis mechanism is more active in a mixed infection as compared to a single one. Furthermore, we performed the MapMan based systematic analysis of DEGs to visualize an unbiased and comprehensive overview of the biological process and their coordinated response to CBCVd, HLVd, and mixed HLVd-CBCVd infections in hop (Figure 5). The appearance of chlorosis in CBCVd infected leaf tissues could be attributed to the reduced expression of genes involved in sucrose and starch biosynthesis, which further leads to an extensive reduction of photosynthesis activity (Figure 5A). Inversely, such a correlation could not be established in a HLVd-CBCVd infected hop plant, but diminished intrinsic chaperone activity was observed (Figure 5C). The chaperones are the main component of the protein quality control mechanism of the cell. The extensive downregulation of genes encoding chaperones suggested a reduction in protein quality control activities, which serve as a protective cellular mechanism to ward off damaged proteins and thus prevent their interference with essential cellular processes. Hierarchical clustering analysis using the expression profile (RPKM value) of the 50 most differentially regulated genes associated with single CBCVd, HLVd, and mixed HLVd-CBCVd infection was performed to validate the reproducibility of the biological replicates (Figure 6). Intriguingly, candidate DEGs were clustered in four groups in all cases, illustrating their relationship, degree of responses, and consistency of biological replicates. The heat map of the 242 common DEGs showed the relative transcripts level in single CBCVd, HLVd, and mixed HLVd-CBCVd infection (Figure S10). 

## 3. Discussion

In the present study, we investigated the dynamic changes in gene expression profile that occur following single-infections of CBCVd or HLVd, and CBCVd-HLVd mixed infections in hop. The different dynamic changes in hop transcriptome were observed for single and mixed-infections, and their correlation with defense-related genes was also diverged. In plants, PTI (pathogen-triggered immunity) and ETI (effector-triggered immunity) constitute the frontline of defense against pathogens as an innate immune system [46]. The PTI is triggered by plasma membrane-bound receptors-like-kinases upon the recognition of the conserved pathogen features known as pathogen-associated molecular patterns (PAMPs), whereas in ETI pathogen-derived effectors or avirulence (Avr) factors are recognized by cytosolic NB-LRR receptors (R proteins) [47]. Eventually, the signal transduction pathway via MAP Kinases cascades leads to the induction of PR proteins, and the generation of reactive oxygen species, ultimately leading to transcriptional reprogramming. The significant inductions of the expression levels of receptor kinases, signaling pathway (mitogen-activated protein kinase and calcium-dependent protein kinases), PR proteins indicates that the innate immunity process was activated as a result of CBCVd, HLVd single and mixed infections in hop. The calcium-dependent protein kinases (CDPKs) are involved in the phosphorylation of the substrates of PTI and ETI pathways to control the transcriptional reprogramming of defense-responsive genes and hypersensitive cell deaths [48]. The CDPKs genes exhibited elevated expression trends and could be correlated to disease development in hop. The Calmodulin (CaM) and calmodulin-like (CML) proteins act as a sensor relay of the Ca^2 +^ signaling pathway and serve as key regulators of pathogen-induced changes in gene expression in plant immune responses [49]. Our analysis clearly showed the activation of CaM and CML genes, indicating the significant role of this signaling molecule in response to CBCVd and HLVd infections in hop. There is no evidence that viroids encode proteins or possess conserved PAMP; therefore, it is improbable that they trigger the ETI-dependent host immune system. Nevertheless, our DEGs data illustrated that the PTI-associated genes were significantly affected in single and mixed infections of CBCVd and HLVd in hop. In the animal system, our current understanding suggests that the double-stranded RNA (dsRNA)-activated protein kinase (PKR) is activated upon binding of dsRNA and involved in viral pathogenesis [50]. This is not surprising considering that RNA-binding proteins (RBPs) serve as key players during viroid infection in host plants. Previous reports have demonstrated that the PKR homolog in plant P58^IPK^ is required by tobacco mosaic virus (TMV) for binding and successful virulence in *Nicotiana benthamiana* and *Arabidopsis thaliana* plants [51]. Similarly, PSTVd utilizes the plant double-stranded-RNA-binding serine-threonine protein kinase (protein kinase- viroid induced; *pkv*) for symptom development [52]. Despite the conceptual relevance of the role of P58^IPK^ and PKV genes in viruses or viroid infections, their elevated expression levels were not observed in either CBCVd or HLVd infected hop plants, which is similar to those reported in the previous studies [26,30,32]. Intriguingly, the elevated expression patterns of several protein kinases were observed, which might be candidate players functioning in the binding of viroid and interaction with PTI pathway in hop plant. However, a better understanding of the mode of action of viroid single-stranded RNA in the activation or suppression of ETI and/or PTI is crucial in the scenario of robust and coordinated manipulation of the host immune system for the strategic control of viroid-infection in plants. 

Our current understanding from several studies suggests that plant hormones contribute redundantly to plant growth, development, and abiotic/biotic stress responses through transcriptional reprogramming [53]. Previously published studies based on gene expression profiles have revealed a complex array of changes in hormone signaling, their biosynthesis and catabolism upon viroid infection, resulting in developmental disorders and the appearance of disease symptoms in the infected plants [26,54]. Our RNA-seq data demonstrated that genes involved in hormone signal transduction pathways such as indole-3-acetic acid (IAA), brassinosteroid (BR), abscisic acid (ABA), ethylene (ET), and jasmonate (JA), were found to be differentially modulated after CBCVd, HLVd single and mixed infections in hop, which is consistent with previous studies showing the alteration of the gene expression of major plant hormone signal transduction pathways upon CBCVd and HSVd infection in hop and cucumber, respectively [32,54]. The importance of another plant hormone such as salicylic acid (SA) arises from its role in the mediation of resistance against biotrophic pathogens, tomato mosaic virus, and citrus exocortis viroid (CEVd) in tomato [55,56]. Interestingly, the gene encoding component of salicylic acid (SA) biogenesis and responses were not found to be induced in either CBCVd, HSVd single, or mixed infection. The lack of induction of genes associated with the SA hormonal pathway observed in our experiment is consistent with previously observed PSTVd-infected tomato, which exhibited the changes of expression of genes associated with the GA and BR signaling pathways, without the alteration of expression of genes linked to SA and JA signaling pathways [25]. Remarkably, among other plant hormones, BR plays a critical role in orchestrating cell division, elongation, growth, and development [57]. The stunting in hop caused by CBCVd could be correlated to the down-regulation of genes involved in the biosynthetic and signaling pathways of BR hormones. These findings corroborate previous studies that have shown the stunted growth in viroid-infected tomato [29] and potato [58] and a similar drift of reduced levels of transcripts associated with BR synthesis enzymes. 

An essential feature of the plant defense response is an impairment of photosynthesis activity by reducing the consistency of stomatal mesophyll conductance to CO_2_ and Rubisco activity, modification in the structure of the chloroplast, and the reduction of photosynthetic reaction centers found in photosystems I and II [59]. Early studies have also presented evidence for the down-regulation of the expression of several genes involved in photosynthesis (photosynthesis-antenna and electron transport genes), chloroplast, and its biogenesis-related genes in viroid-infected plant tissues, which might serve as an active mechanism of the plant defense program [54]. The down-regulation of genes involved in photosynthesis pathways such as the photosystem I and II reaction center, ferredoxin, light harvesting chlorophyll a/b binding proteins and biogenesis of chloroplast such as pentatricopeptide repeat-containing chloroplastic protein, DELLA transcription factors, etc. in CBCVd, HLVd single and mixed infection in hop confirmed the previous reports. The restrictive photosynthesis and chloroplast development repress growth and development in plants [60], which could be one of the causes of the development of symptoms such as chlorosis, stunting, or mosaic in CBCVd and HLVd infected hop plants.

An important part of plant response to pathogen interactions consists of a significant alteration of genes involved in the primary and secondary metabolism [61,62]. Similarly, our transcriptome data revealed that both single and mixed infection caused fluctuation in the expression levels of genes involved in those two metabolic pathways. Our data indicated that viroid infection promoted the expression of genes associated with transporter activity such as ABC transporters to meet the fluctuating demand for transport activities of sugars, amino acids, and secondary metabolites. 

Previous in-depth studies have illustrated that viruses and viroids arrogate or interfere plant ubiquitin-proteasome system (UPS) and heat shock proteins (HSPs), which provides them with novel avenues to promote replications [63], systemic spread, and accumulations [64]. In this context, several presented models indicated that viruses could target endoplasmic-reticulum-associated ubiquitination as well as cytosolic ubiquitin ligases to channelize UPS to new targets, such as argonaute to compromise the host gene silencing machinery [65]. In the present study, genes related to the UPS system were differentially regulated in single CBCVd (15 genes up-regulated and 5 genes down-regulated), HLVd (1 gene up-regulated and 4 genes down-regulated), and mixed infection (5 genes up-regulated and four genes down-regulated). The cellular heat shock proteins (HSPs), components of the protein quality control UPS system, are highly conserved classes of proteins and are involved in preserving cellular homeostasis under different stressful conditions by facilitating folding of nascent proteins, prevention of denatured protein aggregation, translocation, and assembly reactions [66]. It has been shown that the expression level of family homologs of HSPs is massively induced as a consequence of the defense response that frequently accompanies viruses and viroids infection in plants [66,67,68]. Mechanistically, it has been shown that viruses or viroids utilize HSP chaperones for their sheltering, expressions, multiplications, cell-to-cell movement, and the regulation of host defense response directly or indirectly through interactions with DnaJ [69]. Intriguingly, in our study, the expression level of genes encoding different classes of HSPs (HSP33, HSP70, HSP90) were found to be altered as a consequence of single CBCVd, HLVd, and mixed infection, emphasizing their modulatory and interaction roles in viroid infection in hop. 

Several intracellular parasites have evolved remarkable strategies to manipulate the cellular translation machinery via translational reprogramming for their replication and movement [70]. The ribonucleoprotein has long been considered an integral component of translational reprogramming and variation in their composition adds a layer of translational control of selective mRNA for mobilizing host defenses and coordinating innate responses to infection [71]. It is worth noting that the expression of genes that encode ribosomal proteins (the RP regulon) were found to be differentially regulated in the single and mixed-infection of CBCVd and HLVd, suggesting that protein synthesis is significantly disrupted which is likely to be associated with substantial morphological remodeling (stunted growth) in hop.

Mounting shreds of evidence have elucidated that transcription factors (TFs) play important and unique roles as regulators in diverse biological processes, involving developmental processes, in response to biotic and abiotic stresses [72,73,74]. Several families of TFs, such as bZIP (basic-domain leucine-zipper), WRKY, AP2 (APETALA2)/ERF (ethylene-responsive factor), NAM/ATAF/CUC (NAC), MYBs, MYC, and bHLHs (basic helix-loop-helix) are differentially regulated upon viroid infection in plants [26,28]. Similarly, in our study 77, 23, and 30 differentially expressed TFs were identified in hop infected with single CBCVd, HLVd, and their mixed infection, respectively. The differentially expressed TF families identified in this study included MYB, WRK, NAC, zinc finger, bHLH, bZIP, ERF, and WD-40, which further reinforced their regulatory roles in defense response against viroid infection in plants. 

In summary, our analysis of the differential gene expression profile contributes to the ever-expanding insight into the differences between transcriptional changes in hop leaves triggered by single CBCVd, HLVd, and their mixed infection. Overall, the number of DEGs associated with CBCVd infection was significantly higher than those of HLVd and their mixed infection, suggesting that in mixed infection transcriptional gene silencing via gene methylation or direct interaction with hop plant proteins could play a significant role in disease aggressiveness. However, further study is required to elucidate the underlying mechanism and resolve this relationship. 

In conclusion, the extensive transcriptome data generated by our study could serve as a valuable resource, and further, the identification of DEGs will give impetus to research on the identification of molecular markers as well as functional studies working towards strategies for improving viroid resistance in crop plants.

## 4. Materials and Methods

### 4.1. Plant Inoculation and Disease Assessment

Viroid inoculations were performed on the clonally propagated virus and viroid free plants of cv. ‘Celeia’ obtained in a commercial hop nursery of the Institute of Hop Research and Brewing Žalec, Slovenia. Ten plants for each viroid treatment (HLVd, CBCVd, HLVd + CBCVd) were biolistically inoculated [12,75] two months after dormancy using dimeric viroid cDNA prepared from donor plants infected by CBCVd (GenBank KM211547) [32] and HLVd (GenBank X07397) [18]. Inoculum for HLVd + CBCVd treatment was prepared by mixing equimolar quantities of nucleic acids. Each hop plant was inoculated in total with 360 ng cDNA by five shots into leaves using Helios GeneGun (Bio-Rad). Immediately after inoculation plants were put into polyethylene bags to prevent drying of the shot-wound leaf area and transferred to growing chamber conditions at 25 °C and 16h illumination (90 μmol m^−2^ s^−1^ PAR). After one-week, plants were transferred to an isolated test plot under environmental field conditions. Plants of each viroid treatment were additionally isolated by white insect proof mesh (1.6 × 1.6mm, Tenax, Italy) and were grown in 4Lpots as single bine on a string attached to a 3m height wirework. Non-inoculated plants were treated in the same conditions. To confirm viroid infection plants were examined by RT-PCR after 4, 14, and 28- mpi using CBCVd and HLVd specific primers [76,77]. The strand-specific RT-qPCR (ssRT-qPCR) was performed to monitor the titre of HLVd and CBCVd in their individual and mixed infection. At the same time points (mpi) plants were assessed and scored for the presence of leaf malformations (absent: 0; mild: 1; severe: 2), plant stunting and bine cracking (present: 1; absent: 0). During dormancy (7-11 and 20-24 mpi) plants were maintained as a rootstock in the same pots and at the end of second vegetation (31mpi) the green part of each plant was weighted to measure their biomass. 

### 4.2. RNA Extraction, Library Construction and NGS Sequencing 

Total RNA isolation was extracted from approximately 100 mg of mock-inoculated and systematically infected individual CBCVd, HLVd, and CBCVd-HLVd coinfected hop leaves (28 mpi) using a Spectrum^TM^ Plant Total RNA Kit (Sigma-Aldrich, St. Louis, MO, USA) following the manufacturer’s instructions. Briefly, plant material from an individual plant was flash frozen in liquid nitrogen and pulverized. The powder was transferred to lysis solution, vortexed and incubated at 56°C for 5 min. Cellular debris was centrifuged at maximum speed in a benchtop centrifuge and lysate filtrated through filtration column by centrifugation. The binding solution was added to clarify the lysate and mixed by pipetting. RNA was bound to the binding column and DNase digestion was performed by On-Column DNase procedure. A triple washing procedure was followed, and cleaned RNA was eluted in 50 µl of elution solution. Total RNA was quantified by means of absorbance A260/280 using NanoVue spectrophotometer and RNA integrity numbers (RINs) determined by Agilent Bioanalyzer 2100 electrophoresis using RNA 6000 Nano Kit (Agilent Technologies, Santa Clara, CO, USA). RNA samples were stored at –80°C until further processed. 

NGS sequencing of poly-A RNA fraction was performed using the Ion Proton system. First, poly-A mRNA was enriched using Dynabeads^®^ mRNA DIRECT^™^ Micro Kit (Thermo Fisher Scientific, Waltham, MA, USA) following the manufacturer’s procedure using 8 µg of total RNA input from an individual plant. After elution of mRNA from oligo (dT)_25_ Dynabeads the whole amount of isolated mRNA was used directly for RNA-seq library preparation employing Ion Total RNA-Seq kit v2 (Thermo Fisher Scientific, Waltham, MA, USA) following the procedure for low-input RNA-Seq whole-transcriptome. Briefly, mRNA was fragmented using RNase III enzymatic digestion, ligated to Ion Adapters with barcodes and reverse transcribed. After purification, the cDNA was amplified, and further yield and size distribution were assessed by the High Sensitivity DNA Kit (Agilent Technologies, Santa Clara, CO, USA). Three libraries were pooled together in equimolar concentrations and used for the preparation of template-positive Ion PI™ Ion Sphere™ Particles (ISPs) with 200 base-pair average insert libraries following the manufacturer’s procedure. After amplification in Ion OneTouch^™^ 2 System, the enrichment of template-positive particles was performed using Dynabeads^®^ MyOne™ Streptavidin C1 beads on an Ion OneTouch^™^ ES system. The enriched sample was used for sequencing using the Ion PI^™^ Hi-Q^™^ Sequencing 200 Kit on Ion PI™ Chip v3 following the recommended protocol. The sequenced reads were delivered quality and adapter trimmed in unaligned BAM format, which was converted to FASTQ format using the SAMtools. Basic quality control for the sequenced data was performed by FastQC script. Viroid infection status in samples was confirmed by the mapping of reads against the respective sequence of HLVd and CBCVd. The raw RNA-seq were submitted to the Sequence Read Archive (SRA) for public availability. The complete raw RNA-seq datasets for three biological replicates of mock-inoculated, CBCVd infected, HLVd infected, and HLVd-CBCVd coinfected hop plants have been deposited in the NCBI Sequence Read Archive under accession number SRR8775478, SRR8775477, SRR8775467 (mock inoculated); SRR8775476, SRR8775475, SRR8775468 (HLVd infected); SRR8775474, SRR8775473, SRR8775471 (CBCVd infected) and SRR8775472, SRR8775470, SRR8775469 (HLVd-CBCVd), respectively.

### 4.3. Transcriptome Assembly and Identification of Differentially Expressed Genes

The high-quality reads from all the transcriptome datasets were combined, and de novo assembled into contigs termed as unigenes using CLC Genomics Workbench with default parameters settings (K mer = 25). The completeness of the obtained hop transcriptome was evaluated with the BUSCO tool (Benchmarking Universal Single-Copy Orthologs) based on evolutionarily informed expectations of gene content from near-universal single-copy orthologs using the plant odb9 release dataset version [39]. All assembled unigene sequences were compared with hop transcriptome database of the HopBase genomic resources repository (http://hopbase.org/) using MEGABLAST at the typical cut-off E-value of 1.0 x 10^-5^, with similarity level and alignment length more than 95% and 100 bp, respectively. In order to calculate the expression level of each unigenes, the clean reads of each library were mapped to transcriptome reference sequences of HopBase. Furthermore, clean reads were mapped back onto the assembled unigenes, and gene expression levels were estimated by RPKM (Reads per kilobase of exon per million reads mapped) unit using the Expectation-Maximization algorithm of the Trinity software package [44,78]. The obtained count data as an input was exported to DESeq2 R package [79] for determining differential expression from digital gene expression (DGE) data of two groups using a model based on the negative binomial distribution, and resulting *P*-values were adjusted using the Benjamini and Hochberg approach [80] for controlling the false discovery rate (FDR). Genes with FDR adjusted P-value <0.05 and at least a two-fold change (≥2 or ≤−2) were considered as differentially expressed genes (DEGs) between two libraries. A heatmap was constructed using the log-transformed and normalized value of unigenes based on Euclidean distance and complete-linkage methods using the R statistics package heatmap3 [81].

### 4.4. Validation of RNA-Seq Data by Quantitative Real-Time PCR (RT-qPCR)

To examine the reliability of RNA-seq data, two pathogenesis-related (PR) genes, namely, PR1 (PR1 gene family), chitinase-4 like (CH4) and eight randomly selected candidate DEGs were subject to quantitative real-time PCR (RT-qPCR) analysis using specific designed primers (Supplementary Table S8). Aliquots of the total RNA used in RNA-Seq analysis were treated with the TURBO DNA-free™ Kit (Invitrogen, USA) to remove genomic DNA traces and 2 µg of total RNA was used for cDNA synthesis using the Superscript® III First-strand cDNA Synthesis kit (Invitrogen, Carlsbad, CA, USA), following the manufacturer’s instructions. RT-qPCR was performed on the CFX Connect™ Real-Time PCR Detection System (Bio-Rad, Hercules, CA, USA) using 20 µL of reaction mixture containing 10-fold diluted cDNA, 10 µL 2× SYBR green PCR master mix (Applied Biosystems), 10 µM of forward and reverse gene-specific primers (Table S1) under the following amplification condition: Initial denaturation at 95 °C for 3 min, followed by 40 cycles of denaturation at 95 °C for 15 s, annealing at 58 °C for 30 s, and extension at 72 °C for 30 s. At the end of the reaction, the specificity of each primer pair was assessed using a melting curve analysis. The threshold cycles (Ct) of each candidate gene were averaged for triplicate reactions, and the relative transcriptional changes in gene expression levels (fold-change) were calculated by the comparative Ct (2^-ΔΔCt^) [82] using DRH1 (DEAD-box ATPase-RNA-helicase) [83] as an internal reference for gene expression. 

### 4.5. Functional Annotation and Gene Enrichment Analysis

To deduce the putative functions, the assembled sequences were aligned against the NCBI non-redundant (nr) protein database against the Viridiplantae subset of the NCBI nr database via BLASTX with a significance cut-off *E*-value of 1 × 10^−3^. Blast2GO command line tools (version 1.34.0) [84] were used for homology-based functional annotation of the transcripts, which assigned Gene Ontology (GO) terms comprising of three functional groups, such as biological processes, molecular functions, and cellular components to the query sequences. To gain an overview of the gene pathway network and an understanding of the high-level functions and utilities of a biological system, the bidirectional best hit (BBH) method was used for KEGG (The Kyoto Encyclopedia of Genes and Genomes) pathway assignment using the online KEGG Automatic Annotation Server (KAAS) (http://www.genome.jp/kegg/kaas/) [85]. The enrichment analysis was performed to evaluate the enrichment of several GO categories of DEGs compared to all annotated genes. A hypergeometric test equivalent to one-tailed Fisher’s exact with Bonferroni’s correction (FDR ≤ 0.05) was performed using the AgriGO toolkit (Du et al., 2010) to find enrichment of functional categories. The p-value cut off was set at *p* ≤ 0.05 for statistical analysis and all the GO terms qualifying this parameter were visualized using ReviGO [86]. Similarly, a statistical enrichment of DEGs in KEGG pathways was performed using the KOBAS network software [87] with an adjusted *P*-value < 0.05 as the cut-off criterion. Furthermore, the list of gene identifiers and Log_2_ fold change values of DEGs were submitted to the MapMan software [88], and mapping files were created using the Mercator tool (http://mapman.gabipd.org/web/guest/mercator). The mapping file predicted by Mercator was used as an input to the MapMan software for pathway visualization of DEGs involved in hop-viroid interactions. In the case of expression data for duplicated gene identifiers, the lower value of fold-change was used for analysis to avoid an overestimation of the data.

## Figures and Tables

**Table 1:** Infectivity assessment of hop plants (cv. Celeia) following biolistic inoculation of HLVd, CBCVd and HLVd + CBCVd.

Treatments	Before Dormancy (4 mpi)			After First Dormancy (14 mpi)			After Second Dormancy			
							28 mpi			31 mpi
	Leaf Symptom	Plant Stunting	Bine Cracking	Leaf Symptom	Plant Stunting	Bine Cracking	Leaf Symptom	Plant Stunting	Bine Cracking	Green Plant Parts, Weight (g) ^x^
HLVd	0	0	0	0	0	0	0	0	0	135.9 ^a^
CBCVd	0	0	0	0.4	1	0	1	1	0	66.9 ^b^
HLVd + CBCVd	1	0	0	1.7	1	1	2	1	1	27.5 ^c^
Viroid free plants	0	0	0	0	0	0	0	0	0	144.6 ^a^

The data represents mean value of 10 replicates for individual treatment (mock-inoculated, CBCVd or HLVd and CBCVd + HLVd viroid inoculation). Leaf symptoms (absent: 0; mild: 1; severe: 2); Stunting and bine cracking (present: 1; absent: 0) are represented as score. ^x^ Means followed by the same letter were not significantly different at the 5 % level (Duncan’s multiple range test).

## Supplementary Materials

Supplementary materials can be found at https://www.mdpi.com/1422-0067/20/13/3154/s1.
