# Supplementary material for: Evaluation of Disease Severity and Global Transcriptome Response Induced by Citrus bark cracking viroid, Hop latent viroid, and Their Co-Infection in Hop (Humulus lupulus L.)
Source: Int J Mol Sci. 2019 Jun 28;20(13):3154. doi: 10.3390/ijms20133154 (PMC6651264; doi:10.3390/ijms20133154)
Supplement: Supplementary file 1 [file ijms-20-03154-s001.zip › ijms-525108 supplementary final/Table and Figure titles.docx]

**Figure S1:** Strand-specific real-time RT-qPCR analysis for quantification of HLVd and CBCVd in individual and mixed infected hop plants. All samples were normalized to the strand with the higher level (100%) and relative quantities were calculated using target-specific amplification efficiencies. Each column represents the mean ±S.D. of three replicates of single and mixed infected hop plants used for RNA-seq experiments.

**Figure S2:** Characteristics of assembled unigenes. (**A**) Length distribution of assembled unigenes; (**B**) E-value distribution of the BLASTX hits against the nr protein database for each unigene with a cutoff E-value of 1.0 E^−3^; (**C**) Similarity distribution of the top BLASTx hits for each unigene; (**D**) BLASTx top-hit species distribution of unigenes.

**Figure S3:** Determination of functional categories by the Clusters of Orthologous Groups (COG) database. (**A**) COG analysis of assembled in unigenes. (**B**) COG analysis of differentially expressed genes in single HLVd, CBCVd, and mixed HLVd-CBCVd infection. COG category codes: COG category codes are indicated on the X axis and the fraction of unigenes/DEGs in each COG category is shown on the Y axis.

**Figure S4:** Gene ontology (GO) enrichment analysis of upregulated genes arranged in the biological process category (BP), the cellular component category (CC) and the molecular function category (MF), respectively in CBCVd-infected hop plants. The more forward ranking is represented by a redder color.

**Figure S5:** Gene ontology (GO) enrichment analysis of downregulated genes arranged in the biological process category (BP), the cellular component category (CC), and the molecular function category (MF), respectively in CBCVd-infected hop plants. The more forward ranking is represented by a redder color.

**Figure S6:** Gene ontology (GO) enrichment analysis of upregulated genes arranged in the biological process category (BP), the cellular component category (CC) and the molecular function category (MF), respectively, in HLVd-infected hop plants. The more forward ranking is represented by a redder color.

**Figure S7:** Gene ontology (GO) enrichment analysis of downregulated genes arranged in the biological process category (BP), the cellular component category (CC) and the molecular function category (MF), respectively in HLVd-infected hop plants. The more forward ranking is represented by a redder color.

**Figure S8:** Gene ontology (GO) enrichment analysis of upregulated genes arranged in the biological process category (BP), the cellular component category (CC), and the molecular function category (MF), respectively, in CBCVd-HLVd coinfected hop plants. The more forward ranking is represented by a redder color.

**Figure S9:** Gene ontology (GO) enrichment analysis of downregulated genes arranged in the biological process category (BP), the cellular component category (CC), and the molecular function category (MF), respectively, in CBCVd-HLVd coinfected hop plants. The more forward ranking is represented by a redder color.

**Figure S10:** The heat map shows 242 common DEGs in single CBCVd, HLVd, and mixed HLVd-CBCVd infection compared with control in hop. Fold changes for individual transcript is depicted on a log_2_ scale, with a blue-to-dark-red gradient indicating down-and upregulation, respectively.

**Table S1:** Biolistic inoculation and RT-PCR detection of CBCVd and HLVd in hop (cv. Celeia).

**Table S2:** Sequencing statistics for HLVd, CBCVd, HLVd + CBCVd, and control samples.

**Table S3:** Classification statistics for unigenes (UG) and differentially expressed genes [up-regulated (UR) and down-regulated genes (DR)] in CBCVd, HLVd, and CBCVd + HLVd infected hop plant according to KEGG pathway analysis.

**Table S4:** The unigenes differentially expressed between CBCVd infected and mock inoculated hop plants.

**Table S5:** The unigenes differentially expressed between HLVd infected and mock inoculated hop plants.

**Table S6:** The unigenes differentially expressed between HLVd-CBCVd coinfected and mock inoculated hop plants. The raw RNA-seq were submitted to Sequence Read Archive (SRA) for public availability.

**Table S7:** Gene ontology (GO) functional enrichment analysis of differentially expressed genes in CBCVd, HLVd, and CBCVd + HLVd infected hop. **Table S8:** Primers used for qRT-PCR analyses.
