# Supplementary material for: Evaluation of Disease Severity and Global Transcriptome Response Induced by Citrus bark cracking viroid, Hop latent viroid, and Their Co-Infection in Hop (Humulus lupulus L.)
Source: Int J Mol Sci. 2019 Jun 28;20(13):3154. doi: 10.3390/ijms20133154 (PMC6651264; doi:10.3390/ijms20133154)
Supplement: Supplementary file 1 [file ijms-20-03154-s001.zip › ijms-525108 supplementary final/Table S2.docx]

**Table S2:** Sequencing statistics for HLVd, CBCVd, HLVd + CBCVd and control samples

| **Treatment** | | **HLVd** | | | **CBCVd** | | | **HLVd+CBCVd** | | | **Control** | | |
| --- | --- | --- | --- | --- | --- | --- | --- | --- | --- | --- | --- | --- | --- |
| **Biological replicate ID** | | 1-9811 | 2-9814 | 3-9815 | 1-9825 | 2-9828 | 3-9829 | 1-9830 | 2-9832 | 3-9833 | 1-9848 | 2-9849 | 3-9851 |
| **Raw data** |  | | | |  | | |  | | |  | | |
| Reads [M] | | 25.4 | 16.6 | 47.2 | 27.8 | 36.2 | 30.8 | 21.8 | 16.9 | 24.8 | 15.8 | 18.9 | 19.3 |
| Average length [bp] | | 93 | 109 | 115 | 104 | 100 | 129 | 101 | 93 | 105 | 113 | 104 | 105 |
| Amount of data [Gb] | | 2.4 | 1.8 | 5.4 | 2.9 | 3.6 | 4.0 | 2.2 | 1.6 | 2.6 | 1.8 | 2.0 | 2.0 |
| **Clean data** |  | | | |  | | |  | | |  | | |
| Reads [M] | | 23.6 | 16.1 | 45.3 | 26.8 | 34.8 | 29.6 | 21.6 | 16.8 | 24.6 | 14.2 | 17.1 | 17.1 |
| Average length [bp] | | 97 | 111 | 118 | 105 | 102 | 132 | 99 | 91 | 103 | 122 | 111 | 114 |
| Amount of data [Gb] | | 2.3 | 1.8 | 5.3 | 2.8 | 3.6 | 3.9 | 2.1 | 1.5 | 2.5 | 1.7 | 1.9 | 2.0 |
| Number of contigs | | 63,643 | | | 66,358 | | | 50,486 | | | 46,088 | | |
| Maximum Length (bp) | | 13,585 | | | 11,402 | | | 8,744 | | | 7,194 | | |
| Average Length (bp) | | 614 | | | 600 | | | 595 | | | 637 | | |
| Median Length (bp) | | 391 | | | 388 | | | 388 | | | 426 | | |
| N50 (bp) | | 804 | | | 769 | | | 763 | | | 827 | | |
