# Supplementary material for: Evaluation of Disease Severity and Global Transcriptome Response Induced by Citrus bark cracking viroid, Hop latent viroid, and Their Co-Infection in Hop (Humulus lupulus L.)
Source: Int J Mol Sci. 2019 Jun 28;20(13):3154. doi: 10.3390/ijms20133154 (PMC6651264; doi:10.3390/ijms20133154)
Supplement: Supplementary file 1 [file ijms-20-03154-s001.zip › ijms-525108 supplementary final/Table S3.docx]

**Table S3:** Classification statistics for unigenes (UG) and differentially expressed genes [up-regulated (UR) and down-regulated genes (DR)] in CBCVd, HLVd and CBCVd + HLVd infected hop plant according to KEGG pathway analysis

| **KEGG categories** |  | **CBCVd-Infected** | | **HLVd-Infected** | | **CBCVd + HLVd Infected** | |
| --- | --- | --- | --- | --- | --- | --- | --- |
| **Metabolism** | **UG** | **UR** | **DR** | **UR** | **DR** | **UR** | **DR** |
| Carbohydrate Metabolism | 2244 | 68 | 64 | 24 | 18 | 17 | 20 |
| Energy metabolism | 873 | 26 | 54 | 23 | 05 | 14 | 13 |
| Lipid metabolism | 1259 | 73 | 20 | 30 | 07 | 25 | 06 |
| Nucleotide metabolism | 518 | 10 | 10 | 08 | 00 | 01 | 02 |
| Amino acid metabolism | 1381 | 58 | 39 | 48 | 18 | 48 | 10 |
| Metabolism of other amino acids | 514 | 21 | 20 | 09 | 07 | 16 | 07 |
| Glycan biosynthesis and metabolism | 373 | 26 | 04 | 07 | 00 | 01 | 01 |
| Metabolism of cofactors and vitamins | 566 | 11 | 18 | 14 | 00 | 07 | 04 |
| Metabolism of terpenoids and polyketides | 383 | 17 | 11 | 22 | 05 | 07 | 05 |
| Biosynthesis of other secondary metabolites | 962 | 43 | 33 | 20 | 17 | 39 | 13 |
| Xenobiotics biodegradation and metabolism | 440 | 21 | 42 | 09 | 19 | 30 | 18 |
| Enzyme families | 0 | 0 | 0 | 00 | 00 | 00 | 00 |
| **Genetic information processing** |  |  |  |  |  |  |  |
| Transcription | 546 | 05 | 06 | 03 | 02 | 00 | 00 |
| Translation | 1498 | 22 | 75 | 20 | 32 | 17 | 09 |
| Folding, sorting and degradation | 1218 | 33 | 14 | 20 | 03 | 15 | 01 |
| Replication and repair | 781 | 04 | 07 | 02 | 01 | 00 | 03 |
| RNA family | 0 | 00 | 00 | 00 | 00 | 00 | 00 |
| **Cellular Process** |  |  |  |  |  |  |  |
| Transport and catabolism | 1446 | 47 | 22 | 15 | 05 | 11 | 06 |
| Cell growth and death | 1339 | 17 | 26 | 17 | 06 | 21 | 07 |
| Cellular community - eukaryotes | 284 | 04 | 09 | 01 | 00 | 01 | 00 |
| Cellular community - prokaryotes | 163 | 01 | 11 | 04 | 04 | 02 | 05 |
| Cell motility | 111 | 01 | 02 | 01 | 00 | 02 | 00 |
| **Environmental information processing** |  |  |  |  |  |  |  |
| Membrane transport | 180 | 05 | 00 | 02 | 01 | 02 | 01 |
| Signal transduction | 4540 | 127 | 75 | 36 | 39 | 102 | 29 |
| Signaling molecules and interaction | 00 | 00 | 00 | 00 | 00 | 00 | 01 |
| **Unclassified** |  |  |  |  |  |  |  |
| Metabolism | 714 | 14 | 18 | 09 | 20 | 11 | 06 |
| Genetic information processing | 62 | 03 | 00 | 01 | 81 | 00 | 01 |
| Cellular processes and signaling | 136 | 04 | 02 | 00 | 34 | 03 | 00 |
| Viral protein family | 0 | 00 | 00 | 00 | 00 | 00 | 00 |
| Poorly characterized | 83 | 06 | 01 | 03 | 02 | 02 | 01 |
| **Total** | 22614 | 667 | 583 | 348 | 326 | 394 | 169 |
