# Supplementary material for: Evaluation of Disease Severity and Global Transcriptome Response Induced by Citrus bark cracking viroid, Hop latent viroid, and Their Co-Infection in Hop (Humulus lupulus L.)
Source: Int J Mol Sci. 2019 Jun 28;20(13):3154. doi: 10.3390/ijms20133154 (PMC6651264; doi:10.3390/ijms20133154)
Supplement: Supplementary file 1 [file ijms-20-03154-s001.zip › ijms-525108 supplementary final/Table S1.doc]

**Table S1**: Biolistic inoculation and RT-PCR detection of CBCVd and HLVd in hop (cv. Celeia)

| **Viroid treatment^a^** | **Plant no.** | **RT-PCR viroid detection (+/-)** | | | | | |  |
| --- | --- | --- | --- | --- | --- | --- | --- | --- |
|  |  | before  dormancy  ( 4 mpi^b^) | | After  first dormancy  (14 mpi^b^) | | After second dormancy  (28 mpi^b^) | | **Plants sampled for NGS^c^** |
|  |  | **HLVd** | **CBCVd** | **HLVd** | **CBCVd** | **HLVd** | **CBCVd** |  |
| **HLVd** | 1 | + | - | + | - | + | - | + |
|  | 2 | - | - | - | - | - | - | - |
|  | 3 | + | - | + | - | + | - | + |
|  | 4 | - | - | + | - | + | - | - |
|  | 5 | + | - | + | - | + | - | + |
|  | 6 | - | - | - | - | - | - | - |
|  | 7 | - | - | - | - | - | - | - |
|  | 8 | - | - | - | - | + | - | - |
|  | 9 | + | - | + | - | + | - | + |
|  | 10 | - | - | - | - | - | - | - |
| **CBCVd** | 1 | - | - | - | - | - | - | - |
|  | 2 | - | - | - | + | - | + | - |
|  | 3 | - | + | - | + | - | + | + |
|  | 4 | - | + | - | + | - | + | - |
|  | 5 | - | + | - | + | - | + | + |
|  | 6 | - | - | - | - | - | - | - |
|  | 7 | - | + | - | + | - | + | + |
|  | 8 | - | + | - | + | - | + | + |
|  | 9 | - | + | - | + | - | + | - |
|  | 10 | - | - | - | - | - | - | - |
| **HLVd + CBCVd** | 1 | - | - | - | - | - | - | - |
|  | 2 | - | + | - | + | - | + | - |
|  | 3 | - | - | - | + | - | + | - |
|  | 4 | - | + | + | + | + | + | - |
|  | 5 | - | + | - | + | - | + | - |
|  | 6 | + | + | + | + | + | + | + |
|  | 7 | + | + | + | + | + | + | + |
|  | 8 | + | + | + | + | + | + | + |
|  | 9 | - | - | - | - | - | - | - |
|  | 10 | + | - | + | + | + | + | + |

^a^Biolistic inoculation using viroid cDNA

^b^Months post inoculation.

^c^Plants for NGS analysis were sampled in time point 28 months dpi. Three plants (marked with red + ) were used for RNA sequencing.
