# Supplementary material for: Evaluation of Disease Severity and Global Transcriptome Response Induced by Citrus bark cracking viroid, Hop latent viroid, and Their Co-Infection in Hop (Humulus lupulus L.)
Source: Int J Mol Sci. 2019 Jun 28;20(13):3154. doi: 10.3390/ijms20133154 (PMC6651264; doi:10.3390/ijms20133154)
Supplement: Supplementary file 1 [file ijms-20-03154-s001.zip › ijms-525108 supplementary final/Table S7.doc]

**Table S7:** Gene ontology (GO) functional enrichment analysis of differentially expressed genes in CBCVd, HLVd and CBCVd + HLVd infected hop

| **GO ID** | **Ontology** | **Category** | **CBCVd-infected** | | **HLVd-infected** | | **CBCVd + HLVd infected** | | **Number of Unigenes in subgroup** |
| --- | --- | --- | --- | --- | --- | --- | --- | --- | --- |
|  |  |  | **Number of DEGs** | **p-value** | **Number of DEGs** | **p-value** | **Number of DEGs** | **p-value** |  |
| **GO:0044238** | primary metabolic process | P | 352 | 0.23 | 144 | 0.43 | 143 | 0.95 | 8995 |
| **GO:0005975** | carbohydrate metabolic process | P | 93 | 1.30E-17 | 28 | 0.00041 | 32 | 0.00015 | 866 |
| **GO:0009987** | cellular process | P | 413 | 0.95 | 164 | 0.097 | 150 | 1 | 11684 |
| **GO:0019538** | protein metabolic process | P | 98 | 1 | 54 | 0.09 | 35 | 1 | 4009 |
| **GO:0006464** | protein modification process | P | 9 | 1 | x | x | x | x | 1474 |
| **GO:0006629** | lipid metabolic process | P | 45 | 0.016 | 19 | 0.08 | 21 | 0.084 | 841 |
| **GO:0009058** | biosynthetic process | P | 211 | 0.097 | 88 | 0.21 | 79 | 0.93 | 5118 |
| **GO:0009056** | catabolic process | P | 38 | 0.96 | 15 | 0.92 | 21 | 0.72 | 1307 |
| **GO:0015979** | photosynthesis | P | 27 | 1.40E-09 | 18 | 5.20E-10 | 24 | 3.40E-14 | 162 |
| **GO:0006810** | transport | P | 68 | 0.6 | 17 | 0.99 | 29 | 0.78 | 1846 |
| **GO:0051179** | localization | P | 69 | 0.68 | 08 | 099 | 29 | 0.84 | 1922 |
| **GO:0019725** | cellular homeostasis | P | 22 | 3.10E-06 | 6 | 0.0062 | 9 | 0.0052 | 174 |
| **GO:0006412** | translation | p | 76 | 0.0035 | 42 | 0.00016 | 28 | 0.36 | 1445 |
| **GO:0009605** | response to external stimulus | P | 11 | 0.93 | 7 | 1 | 8 | 0.98 | 429 |
| **GO:0003824** | catalytic activity | F | 664 | 4.20E-63 | 276 | 1.30E-27 | 333 | 1.50E-39 | 9638 |
| **GO:0016740** | transferase activity | F | 200 | 1.40E-10 | 64 | 0.058 | 130 | 5.00E-17 | 3321 |
| **GO:0016787** | hydrolase activity | F | 181 | 1.10E-05 | 86 | 2.50E-05 | 73 | 0.083 | 3468 |
| **GO:0005488** | binding | F | 695 | 7.70E-49 | 276 | 1.10E-14 | 376 | 9.40E-44 | 11258 |
| **GO:0016301** | kinase activity | F | 89 | 0.0015 | 18 | 0.96 | 77 | 6.20E-14 | 1641 |
| **GO:0016772** | electron carrier activity | F | 23 | 0.0014 | 10 | 0.021 | 10 | 0.043 | 294 |
| **GO:0005215** | transporter activity | F | 92 | 5.40E-06 | 31 | 0.07 | 43 | 0.0016 | 1477 |
| **GO:0008135** | lyase activity | F | 34 | 0.0001 | 29 | 2.50E-10 | 23 | 6.70E-06 | 430 |
| **GO:0005198** | structural molecule activity | F | 77 | 1.40E-16 | 41 | 5.30E-13 | 23 | 0.0024 | 659 |
| **GO:0016020** | membrane | C | 179 | 0.02 | 64 | 0.52 | 63 | 0.9 | 4068 |
| **GO:0005737** | cytoplasm | C | 134 | 1 | 54 | 1 | 43 | 1 | 6822 |
| **GO:0005623** | cell | C | 394 | 1 | 164 | 1 | 147 | 1 | 15217 |
| **GO:0043226** | organelle | C | 202 | 1 | 92 | 1 | 64 | 1 | 8155 |
| **GO:0005840** | ribosome | C | 73 | 1.70E-19 | 41 | 4.20E-16 | 23 | 0.00012 | 524 |
| **GO:0009536** | plastid | C | 7 | 1 | x | x | x | x | 2965 |
| **GO:0005618** | cell wall | C | 15 | 0.56 | 6 | 0.61 | 7 | 0.58 | 403 |
| **GO:0005829** | nucleoplasm | C | 10 | 0.15 | 9 | 0.0026 | 7 | 0.044 | 177 |
| **GO:0005886** | nucleus | C | 66 | 1 | 32 | 0.95 | 28 | 1 | 2621 |
