# Supplementary material for: Evaluation of Disease Severity and Global Transcriptome Response Induced by Citrus bark cracking viroid, Hop latent viroid, and Their Co-Infection in Hop (Humulus lupulus L.)
Source: Int J Mol Sci. 2019 Jun 28;20(13):3154. doi: 10.3390/ijms20133154 (PMC6651264; doi:10.3390/ijms20133154)
Supplement: Supplementary file 1 [file ijms-20-03154-s001.zip › ijms-525108 supplementary final/Table S8.doc]

**Table S1:** Primers used in qRT-PCR analysis

| **S. No.** | **Primer name** | **Sequence (5´-3´)** | **GC %** | **Tm** | **Target name** |
| --- | --- | --- | --- | --- | --- |
| 1 | qRT_MYB_F | CAGAGAAGTTAGAAGGAAGATCAG | 41 | 58.61 | MYB transcription factor |
| 2 | qRT_MYB_R | CTTGCCATTATCACATGCCA | 45 | 59 |  |
| 3 | qRT_RC_F | AAGATTAGACTTGGAAGAGGGTG | 43 | 60 | Golgi snap receptor complex member 1-1 |
| 4 | qRT_RC_R | GGCTGCTAACATTGCTGAG | 52 | 59 |  |
| 5 | qRT_GPE_F | TAAGCCTCCTAAAGCAATTCGT | 40 | 60.27 | Glucose-6-phosphate 1-epimerase |
| 6 | qRT_GPE_R | ACCTGTTTCTAGCAAATCCGT | 42 | 60.15 |  |
| 7 | qRT_ST_F | AGAGCGTTGAGTTTATGCCGT | 47 | 62.88 | Bidirectional sugar transporter n3 |
| 8 | qRT_ST_R | TACCATACATGAGCCACATAACAG | 41 | 60.73 |  |
| 9 | qRT_SRK_F | ATCCCACCCTTGTAAACCC | 52 | 59.39 | Somatic embryogenesis receptor kinase 1 |
| 10 | qRT_SRK_R | ACCAGACAAAGCAGCATTTCC | 47 | 61.93 |  |
| 11 | qRT_CHI_F | AACCCAAGCAAGAAATACTACGG | 43 | 60.45 | Chitinase 4-like protein |
| 12 | qRT_CHI_R | TGAACATTCTCCATCCAGAACC | 45 | 61.62 |  |
| 13 | qRT_PR1_F | GAAGGTACCCTTATTGTTGTTGCA | 50 | 60.37 | Pathogenesis-related protein PR1 |
| 14 | qRT_PR1_R | GTTTGCGGGCACTACACTCA | 41 | 62.07 |  |
| 15 | qRT_CEL_F | TCTCTTGCCTCTCGAACAG | 55 | 59.19 | Cellulase protein |
| 16 | qRT_CEL_R | ATCACCAAAGAAACCATTGCC | 42 | 59.87 |  |
| 17 | qRT_BC_F | CGTAGTTGGAGATAGCCTAGTG | 50 | 59.94 | Blue copper protein |
| 18 | qRT_BC_R | CGCCACAGATGAAGTAATGAG | 47 | 59.27 |  |
| 19 | qRT_TR_F | TACGCCAATCCTGACCGA | 55 | 60.83 | Thioredoxin h-type-like |
| 20 | qRT_TR_R | CCACTCCTTAGCAACATCCT | 50 | 59.57 |  |
| 21 | qRT_DRH1_F | ATTCCCACTAAGCGTCAAACC | 50 | 61.11 | DEAD-box ATPase-RNA-helicase |
| 22 | qRT_DRH1_R | ATCTGCTCTAACCTCCTCTGT | 45 | 60.22 |  |
